# An Orthopaedic Robotic-Assisted Rehabilitation Method of the Forearm in Virtual Reality Physiotherapy

**DOI:** 10.1155/2018/7438609

**Published:** 2018-08-01

**Authors:** Miguel A. Padilla-Castañeda, Edoardo Sotgiu, Michele Barsotti, Antonio Frisoli, Piero Orsini, Alessandro Martiradonna, Cristina Laddaga, Massimo Bergamasco

**Affiliations:** ^1^Laboratory of Perceptual Robotics, Pisa, Scuola Superiore Sant' Anna, Italy; ^2^Instituto de Ciencias Aplicadas y Tecnología, Universidad Nacional Autónoma de México, México City, Mexico; ^3^Microfabrication and Exploratory Nanotechnology, International Iberian Nanotechnology Laboratory, Braga, Portugal; ^4^Research Center “E.Piaggio”, University of Pisa, Pisa, Italy; ^5^Adult Rehabilitation Centre “Luciana Segenni” USL5, Fornacette, Italy

## Abstract

The use of robotic rehabilitation in orthopaedics has been briefly explored. Despite its possible advantages, the use of computer-assisted physiotherapy of patients with musculoskeletal injuries has received little attention. In this paper, we detailed the development and evaluation of a robotic-assisted rehabilitation system as a new methodology of assisted physiotherapy in orthopaedics. The proposal consists of an enhanced end-effector haptic interface mounted in a passive mechanism for allowing patients to perform upper-limb exercising and integrates virtual reality games conceived explicitly for assisting the treatment of the forearm after injuries at the wrist or elbow joints. The present methodology represents a new approach to assisted physiotherapy for strength and motion recovery of wrist pronation/supination and elbow flexion-extension movements. We design specific game scenarios enriched by proprioceptive and haptic force feedback in three training modes: passive, active, and assisted exercising. The system allows the therapist to tailor the difficulty level on the observed motion capacity of the patients and the kinesiology measurements provided by the system itself. We evaluated the system through the analysis of the muscular activity of two healthy subjects, showing that the system can assign significant working loads during typical physiotherapy treatment profiles. Subsequently, a group of ten patients undergoing manual orthopaedic rehabilitation of the forearm tested the system, under similar conditions at variable intensities. Patients tolerated changes in difficulty through the tests, and they expressed a favourable opinion of the system through the administered questionnaires, which indicates that the system was well accepted and that the proposed methodology was feasible for the case study for subsequently controlled trials. Finally, a predictive model of the performance score in the form of a linear combination of kinesiology observations was implemented in function of difficult training parameters, as a way of systematically individualising the training during the therapy, for subsequent studies.

## 1. Introduction

Musculoskeletal disorders or lesions in conjunction are one of the leading causes of chronic disability around the world. For example, in the United States, orthopaedic surgery is one of the first causes of medical visits and physical therapy is one of the nonmedicated treatments [1].

Among orthopaedic injuries, wrist fractures had a high incidence in the elderly population in 2001 [2]; forearm fractures of the distal radius are the most common in humans [3]. Patients with a distal radial fracture must require staying out of work around 67 days up to 20 weeks for recovery, what poses relevant economic and social implications. Indeed, at the moment of suffering the radius injury, more than half of the patients are currently employed [4]. A study for evaluating the relationship of pain, occupational performance, and quality of life in a women population after upper limb fractures indicates that half of the reported problems were related with productivity, almost 40% with self-care, and 10% with leisure [5].

Although less frequent, elbow fractures might lead to severe limitations of the forearm, affecting its fundamental role in placing and supporting the hand in the space and as a stabiliser [6]. Elbow fractures can occur at the distal humerus, the proximal radius, or the proximal ulna. Such injuries result in considerable variability of postfracture symptoms (swelling, pain, and loss of motion) and might lead to functional disability [4, 6] because these joints should hold mobility, stability, strength, and absence of pain [7, 8]. Loss of motion of the elbow may affect essential independent functions in daily life activities, including personal care, mobility, eating, or even walking safely with aids especially for the elderly [8].

The treatment depends on the lesion severity; if the fracture is stable and without dislocation of fragments, a cast or a splint made of thermoplastic material is used for external immobilisation of the lesion area. An unstable and dislocated fracture requires a surgical intervention of reduction and stabilisation and the following immobilisation with a cast or a splint [8, 9]. In any case, after the immobilisation period, an early rehabilitation treatment consisting of exercise physiotherapy must start as early as possible to have a positive recovery of the forearm motion. Four aspects have been suggested as crucial factors for recovery functional movement in patients with fractures affecting the forearm joints: the number of therapy visits [10], the intensity and individualising level of the therapy, the adherence to the postoperative treatment [10], and the objective and continuous monitoring of the patient evolution during the intervention. Thus, on the one hand, the need for reducing the duration of the postoperative treatment for functional restoration is a crucial factor for many patients. On the other hand, the need for therapists for incrementing the number of attending patients with a better understanding of the progress of the patients motivates the research of treatment strategies that would optimise the type, intensity, and duration of the treatment according to the patient's condition.

An approach based on rehabilitation robotics with physiotherapy games would be a valuable tool for rendering the physiotherapy process more efficient. However, most of the current research in rehabilitation robotics focuses on neurorehabilitation for patients with lesions at the central nervous system (CNS), with more emphasis on stroke patients and with lesser extent in patients with other neural injuries, such as in the spinal cord. Exoskeletons [11–14] or end-effector robots [15] in combination with virtual reality (VR) serious games [16–19] have shown advantages for the neuromotor rehabilitation of upper limbs [20–23]: to mention, higher intensity of the training, higher level of motor control of the joints, longer duration, and more number of the sessions [13, 24], which can give variable assistance [25] or resistance [26] force feedback and may provide kinesiology information of the patient performance that facilitates the evaluation during the treatment [23], among others.

Unfortunately, despite the possible benefits, the high incidence of musculoskeletal injuries, and the current demand for faster and better physical therapies, the use of robotics for orthopaedic rehabilitation remains practically uncovered, and the research in this field is quite scarce, in comparison with neurorehabilitation systems [27].

The main difference between neuromotor and orthopaedic rehabilitation relies on the clinical goal of the recovery. For neurologic patients (with injuries at the level of the CNS), the primary goal is to achieve a cortical reorganisation which could lead to the restoration of motor functionalities [28, 29]. Instead, for orthopaedic patients (with musculoskeletal lesions but without cognitive impairments), the primary goal of any system should be the restoration of functional ranges of motion, muscular strength recovery, and pain reduction [5, 6, 8, 9, 30]. Robotic neurorehabilitation focuses on the mobilisation of the limbs through complex multijoint movements (reaching, grasp, and bimanual coordination, among others) and neuroplasticity stimulation with cognitive task assignment [17]. After a period of immobilisation, robotic rehabilitation in orthopaedics should focus on mobilisation of single joints within moderate increments [8, 9, 30].

Due to its severity, the treatment of a musculoskeletal lesion, such as elbow fracture, must be carried out with precaution and special care must be taken during all the phases of the intervention [8]. First, if the fracture is stable and without dislocation of fragments, a cast or a splint made of thermoplastic material is used for external immobilisation of the lesion area. An unstable and dislocated fracture requires a surgical intervention of reduction and stabilisation and the following immobilisation with a cast or a splint [31]. Aside from ensuring stabilisation of fragments, immobilisation aids to decrease pain and swelling and importantly prevents radiological deformity [32]. However, a common complication of postsurgery immobilisation is the development of joint stiffness and consequently long-term loss of range of motion [33]; in the case of the elbow, up to 25% of distal humerus fractures result in elbow stiffness [34]. For this reason, the rehabilitation must start as early as possible, immediately after the absence of severe pain, oedema, or instability of fragments [31]. Besides preventing contracture formation and posttraumatic rigidity, this facilitates the recovery of functional range of motion (RoM) and muscular strength [6].

The rehabilitation relies on the intensive practice of isolated movements [6, 8], actively performed by the patient or passively assisted by the therapist. Usually, the orthopaedic rehabilitation consists of three subsequent set of exercises: passive, active-assistive, and active. Passive exercises consist of the manual mobilisation of the patients' articulations performed by the therapist. Then, active-assistive exercises are performed when patients' muscles are still feeble and they have difficulties to perform the exercises independently. Finally, active exercises are performed in the latest phase (usually the longer) of the rehabilitation when the patient can move without external assistance [8, 9]. Mobilisation must be done gradually from passive to active movements of the single joints, starting from moderate movements within reduced RoM and mild working loads, especially at the early days after immobilisation, up to functional RoM and higher loads for strength recovery. This process is challenging for the therapist: it must focus on functional motion recovery and avoiding at the same time any aggressive movement that may provoke postinjury complications, for example, trauma to the arm's brachialis muscle and the elbow joint capsule due to forced passive manipulation of the elbow [33].

With these motivations, some devices have been designed specifically for wrist [15, 35–38] and elbow joints [36, 39], but to date, they have not been integrated with physiotherapy applications specifically designed for such patients. In particular, Vanderniepen et al. [39] presented an elbow orthosis intended explicitly for orthopaedic rehabilitation of the elbow, with an adaptable compliance mechanism designed for slow and constrained motions in a single DoF for protection of the patient and then for extending its device to an exoskeleton considering the shoulder [14]. Both apparatus seem suitable for orthopaedic rehabilitation of the upper limb. However, they did not include any integration with specific software for this purpose, and they did not report any experience with patients.

On the contrary, even if some of the rehabilitation robots reported for neurorehabilitation could have the potential use in orthopaedic rehabilitation, the adopted human-robot interaction schemes and assisted physiotherapy approaches of such systems cannot be directly applied for the needs of orthopaedic patients [40, 41]. At most, they should be used with precaution, but in any case, it is not clear if neurorehabilitation approaches would be valid for orthopaedic lesions, given the noticeable different clinical goals [41].

Up to the best of our knowledge, nowadays, the work reported by Schwickert et al. [42] is the only study detailing the use of robotic-assisted rehabilitation in orthopaedics with patients for proximal humerus fractures in virtual environments using the ArmeoSpring system (Hocoma AG, Zurich, Switzerland). The study consisted of an uncontrolled intervention of eight case series, with robotic sessions 2-3 times per week during 2–4 weeks in robotic sessions in combination with manual therapy; some single- to multijoint movements were tested with patients playing in goal-oriented game scenarios and with difficulty increments manually adjusted. Their results indicated that the system was safe and the treatment was feasible. However, the study gives no details regarding the specificity of the games for the characteristics of orthopaedic physiotherapy and nor the adopted criteria of how to adapt the difficulty levels during the sessions, which is not a straightforward aspect but still an open-research aspect. Thus, more research should be done both for the development of new devices and software-based physiotherapy methodologies for orthopaedic patients and for studying the feasibility, validation, and effectiveness of such developments through clinical experimentation with patients.

On these bases, we developed a robotic system for the orthopaedic rehabilitation of the upper limb, which integrates a novel methodology for assisted physiotherapy in VR serious games. The system consists of four modules: (1) a robotic rehabilitation device (PERCRO-BRANDO); (2) VR serious games for motion task execution of the forearm; (3) the therapist graphic interface; and (4) and a task difficulty adaptation module based on the monitored patient kinesiological performance through time.

The BRANDO robot consists of the integration of a 6-DoF end-effector-based robotic arm and a passive arm, mounted together in a common platform. Three of the 6 DoFs are actuated allowing the mobilisation of the elbow and shoulder joints. The three passive DoFs correspond to a 3-DoF gimbal for passive motions of the wrist joint. The passive arm provides full gravity compensation of the upper limb, to aid the patient to perform movements without carrying its weight, which is often considered a useful configuration in manual physiotherapy [6]. The VR serious game applications were conceived for assigning isolated motion tasks of pronation/supination (PS) or flexion/extension (FE) movements within constrained RoM and moderate but incremental strength loads, within motivating game scenarios at different levels of difficulty. With the user-friendly graphical interface, the therapist can evaluate the current mobility of the FE and PS, modify the difficulty parameters of the exergames, and monitor the kinetic progress during the sessions. Finally, the task difficulty adaptation module allows to help the therapists to tailor the physical demanding of exercises based on the monitored kinesiological information and through the estimation of the performance score through a regression model, previously calibrated experimentally.

To the best of our knowledge, the system described in this paper is the first one designed that combines these four components in a single framework on the basis of the specific needs of orthopaedic rehabilitation of the upper limb, in particular for the recovery of strength and range of motion of elbow movements affecting the forearm. With the aim of studying the suitability of the proposed methodology with the system, we carried out two experiments: first in healthy subjects and second in a group of patients under orthopaedic rehabilitation of the forearm. Firstly, through the analysis of electromyographic surface signals (sEMG) placed at the arm muscles of the two healthy subjects practising with the system under the same conditions conceived for patients, we verified the capability of the method of assigning different working loads without physically overloading the patients until their muscular fatigue. Secondly, we tested the system on ten patients (9 performing PS movements and 7 performing PS and FE movements) confirming not only the acceptance by the patients but also its functional ability in delivering customised demanding levels of exercises systematically and safely. In conclusion, the proposed system is a suitable platform for carrying out more clinical studies towards the validation and effectiveness of robotic-assisted orthopaedic rehabilitation of the forearm and paves the way to the design of new therapeutic interventions for the rehabilitation of other upper limb fractures.

The rest of the paper is organised as follows: Section 2 presents the development of the full rehabilitation system.  Section 3 presents the assessment of the system performance with the healthy volunteers and the feasibility and acceptance assessment by patients.  Section 4 presents the discussion, and Section 5 the conclusions.

## 2. The BRANDO Rehabilitation System

Our system called BRANDO consists of the robotic device integrated with a control scheme for active/passive rehabilitation of the upper limb, through VR exercising gaming scenarios. With this system, we propose a new methodology of assisted physiotherapy for the orthopaedic rehabilitation of the forearm, as detailed in this section.

### 2.1. Robotic Rehabilitation Device

**Figure 1:**
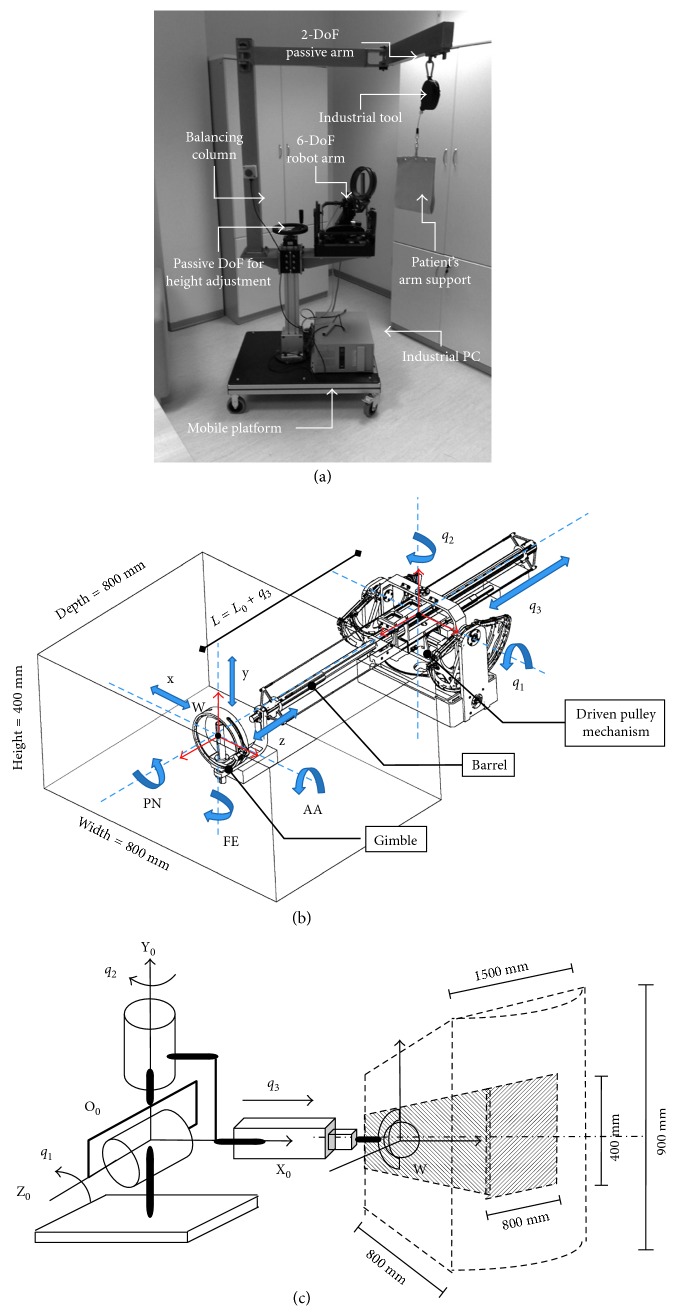
BRANDO rehabilitation robotic system consisting of a 6-DoF robot device mounted on the passive mobile platform, with a passive handle attached at the end-effector. (a) The full final system. (b) The mechanism of the 6-DoF robotic arm. (c) Conical workspace of the robot, with a minimum reachable square workspace of 400 × 800 × 800 mm.

The BRANDO system (Figure 1) consists of a haptic interface in the form of a 6-DoF robotic arm with 3 actuated DoFs [43], enhanced with a 3-DoF passive gimbal for the patient's hand and mounted in a passive mechanism for giving support to the upper limb [44].

The haptic interface consists of a 3 actuated DoF end-effector (EE) mechanism (Figure 1), kinematically equivalent to 2 orthogonal, one incident rotational joints (*q*_1_ ± 25° and *q*_2_ ± 45°) and one prismatic joint (*q*_3_ = 0.630 mm) that drives a barrel along a third incident axis. The following direct kinematic equations define the tracking of the EE position in space:

(1) $$\begin{array}{c} x=L\;\cos\left(q_{1}\right)\;\cos\left(q_{2}\right),\\ y=L\;\sin\left(q_{1}\right)\;\cos\left(q_{2}\right),\\ z=-L\;\sin\left(q_{2}\right), \end{array}$$

where *L*=*q*_3_+*L*_o_ is the length of the barrel's displacement, with *L*_*o*_=441 mm being the minimum displacement.

Two DoFs are actuated by means of a differential transmission, composed of 2 capstans acting on a commonly driven pulley. The differential transmission was designed to allowing high kinematic isotropy along *x*- and *z*-directions and high regularity of the diagonal elements of the corresponding mass matrix of the mechanism. Three brushed DC motors are used for actuation: two grounded motors for reducing the amount of moving mass and the third motor for providing the translational motion of the barrel. No reduction gear was employed to minimise the backlash. The mechanism allows backdrivability of the DoFs with low friction perceived at the EE. Two weights are fixed to the rear of the barrel to counterbalance its weight in the central position of the workspace.

A passive gimbal with three spherical DoFs was mounted at the EE to allow the patient to handle the interface (wrist position W). The gimbal allows the tracking of the flexion/extension (FE) and abduction/adduction (AA) of the wrist and the pronation/supination (PS) of the elbow of the patient. It includes two buttons to let the patient trigger simple commands during the exercises.

The passive mechanism consists of one prismatic joint to adjust the height of the interface to the vertical position of the hand of the patient and a balancing column to mount a 2-DoF passive arm. The mechanism allows compensating the weight of the forearm through an ergonomic base supported by an industrial tool (with a maximum payload of 4 kg). A mobile platform mounts the full mechanism and allows placing the full device stable in the clinical room.

The full BRANDO system resulted in the optimal configuration shown in Figure 1. The configuration allows the patient to place comfortably in a seated posture and perform upper limb movements by placing the wrist within a minimal workspace of 400 × 800 × 800 mm up to an approximately 900 × 800 × 1500 mm conic workspace, under maximum continuous force feedback of 10 N up to a peak force of 20 N.

### 2.2. Patient's Upper Limb Tracking and Modelling

**Figure 2:**
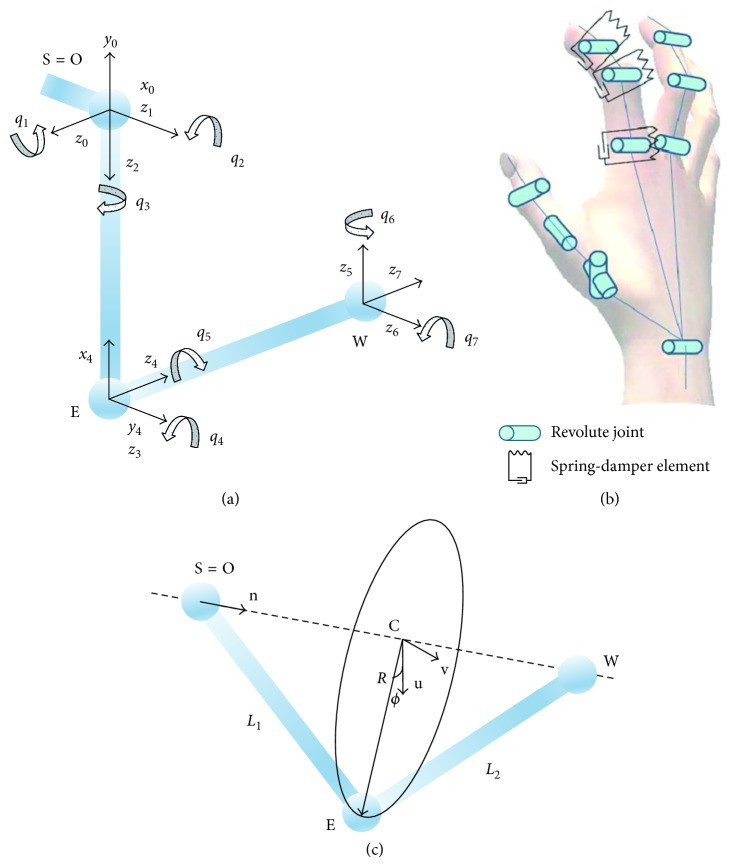
A simulation model of the upper limb. (a) 7-DoF kinematic model of the arm. (b) 17-DoF kinematic model of the hand, with the physical interpretation as a multirigid body system with spring-damper joints. (c) Elbow position as a function of the swivel angle *ϕ*.

The system integrates a virtual reality model of the human upper limb [45], consisting of a multibody rigid dynamic system with 7 DoFs for the arm (Figure 2) and 17 DoFs with 18 links for the hand (Figure 2), in the form of revolute joints [46]. We implemented the model in the *XVR* software for VR (VRMedia s.r.l., Pisa, Italy) and C++ using the *PhysX SDK* (Nvidia, USA) for the physics engine simulation.

The tracking of the patient's movements is done by the estimation of the joint angles of the patient limb (*q*_1_ to *q*_7_), given the estimated positions **W**, **E**, and **S** using the analytical inverse kinematic solution reported in [47]. The shoulder position **S** is estimated from the starting posture of the patient sitting and with the elbow half flexed. The estimation of the elbow position **E** is according to the physical interpretation illustrated in Figure 2, where due to the redundant mechanism of the upper limb, even fixing **W**, the elbow **E** is still free to swivel on an arc with origin **c** lying in a plane that is orthogonal to the composed axis from the wrist to the shoulder **S** [47]. Given the local coordinates frame by the vectors **n**, **u**, and**v**, it is possible to geometrically estimate **E** in function of the swivel angle *ϕ*, as detailed in [47], where **n** is the normal vector of the wrist-shoulder axis, **u** is the projection of the arbitrary unit vector **a** on the plane which corresponds to *ϕ*=0, *L*_1_ is the length of the upper arm, and *L*_2_ is the length of the forearm.

For the game scenarios presented in this paper, we set up the swivel angle to two possible training postures, for the games in Section 2.4: (1) with the arm adducted at 10° approximately and (2) with the arm abducted at about 80°. Both configurations were estimated empirically with an extendable goniometer (Lafayette Instrument Co, Inc.; model 01135) during preliminary tests. For the first posture, variations of the swivel angle were minimised by instructing the patient to avoid shoulder movements or by fixing the upper arm to the trunk with an elastic bandage; for the second posture, the swivel angle was maintained by placing the patient's upper arm in the arm support of the device. Possible singularities occurring at the elbow fully extended were procedurally detected and corrected at runtime using the gradient information of the position in joint space.

### 2.3. Control Scheme for Patient-Robot Interaction

**Figure 3:**
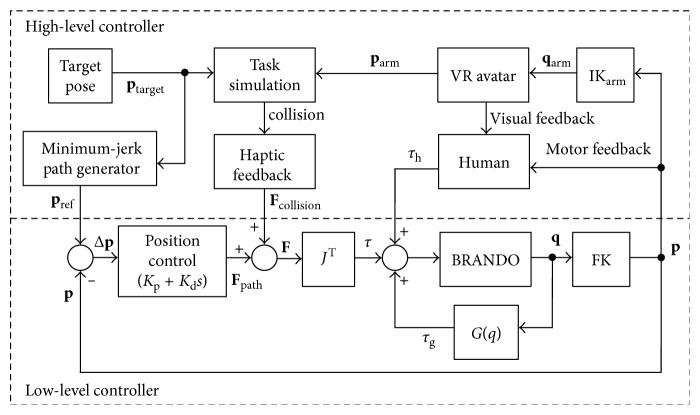
Control architecture for the task simulation through the patient's movements. *G*(*q*) is the gravity model to compute the gravity compensation torque; FK is the direct kinematics block; IK_arm_ is the inverse kinematics algorithm of the patient arm. *τ* and *τ*_g_ are the torque supplied by the motors given the control position and gravity compensation, respectively.

The control scheme was implemented at two levels (Figure 3). An industrial PC with a real-time operating system (xPC Target by MathWorks©) runs the low-level controller. This controller estimates the EE position in space given the **q** angular positions of the robot joints, executes the gravity compensation (G(**q**)) to avoid carrying the load of the robot arm, and performs the proportional-derivative (PD) position control for placing the EE in space. The high-level controller runs on a graphics workstation. This controller runs (1) the inverse kinematics for tracking the patient's movements; (2) the virtual reality games; (3) the target pose selection and the minimum-jerk reference path generator to the target position; and (4) the haptic feedback rendering. The communication between low-level and high-level controllers is executed via UDP.

The motion tasks consist of placing the current EE position (**p**) until touching a virtual object T at different target positions in the game. The interaction loop consists of three phases: (1) selecting T and computing the corresponding target position (**p**_**T**_); (2) exerting haptic forces (**F**_path_) tending to place **p** at a reference position (**p**_ref_), by following a reference minimum-jerk trajectory *R*; and (3) exerting collision forces (**F**_collision_) when the virtual hand (**p**_hand_) touches T.

**Figure 4:**
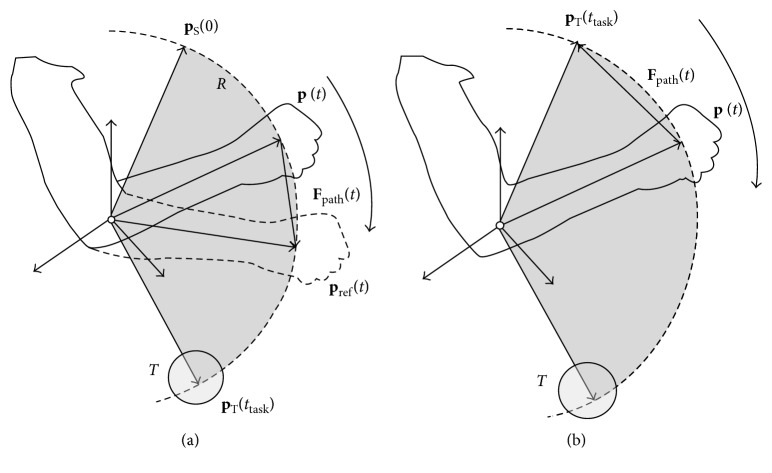
Force feedback scheme to the patient during the execution of elbow joint movements. (a) The assisted mode is activated during passive or active-assisted exercising by aiding the patient to follow a trajectory from the starting to the target positions, where the target position corresponded to the current target object in the VR scenario. (b) The resistive mode is activated during assisted exercising, pushing the end-effector towards the opposite side of the patient movement.

The trajectory R from the starting position **p**_**s**_ to **p**_**T**_ is generated in real time given the polynomial time law reported in [25] (Figure 4(a)):

(2) $$\begin{array}{c} p_{\mathrm{ref}}\left(t\right)=p_{S}+\left(10{\left(\frac{t}{t_{\mathrm{task}}}\right)}^{3}-15{\left(\frac{t}{t_{\mathrm{task}}}\right)}^{4}+6{\left(\frac{t}{t_{\mathrm{task}}}\right)}^{5}\right)\left(p_{T}-p_{S}\right), \end{array}$$

where *t* is the current time and *t*_task_ is the assigned time for completing the task. The equation assumes that the velocity and acceleration are zero at the beginning and the end of *R*. *R* could be linear or circular, depending on the motion task: linear for reaching or circular for single-joint motion tasks.

Positioning the EE is done by a PD controller given the proportional (*K*_p_) and derivative control coefficients (*K*_d_), as follows:

(3) $$\begin{array}{c} F_{\mathrm{path}}\left(t\right)=K_{p}\Delta p+K_{d}\Delta \dot{p},\\ \Delta p=p_{\mathrm{ref}}\left(t\right)-p\left(t\right). \end{array}$$

The therapist can manually adjust the values of *K*_p_, *K*_d_, and *t*_task_. The value of *K*_p_ is within the range [0, 1]Nm, while for stability, *K*_d_ is proportionally computed to keep the ratio *K*_p_/*K*_d_ always constant; such a ratio has been determined empirically during the preliminary tests. As illustrated in Figure 4(a), during the passive or active-assisted training, the resulting haptic positioning forces are exerted as an aid to the patient to follow the reference trajectory towards the target position. For the active exercise, the generated force field is applied to the patient movement by pushing the EE towards the opposite side of the current target object T [26] (Figure 4(b)). The resulting resistance force is generated by placing **p**_**T**_ at the opposite limit position of the training workspace with respect to T. This solution provided opposite resistive forces to the patient during active training, proportional to the amount of elbow joint excursion.

Finally, for safety reasons, manually locking the end-effector position by the therapist is also possible at any moment, which is useful for recording the motion limits of the patient at the beginning of the session.

### 2.4. Virtual Reality Gaming Scenarios

**Figure 5:**
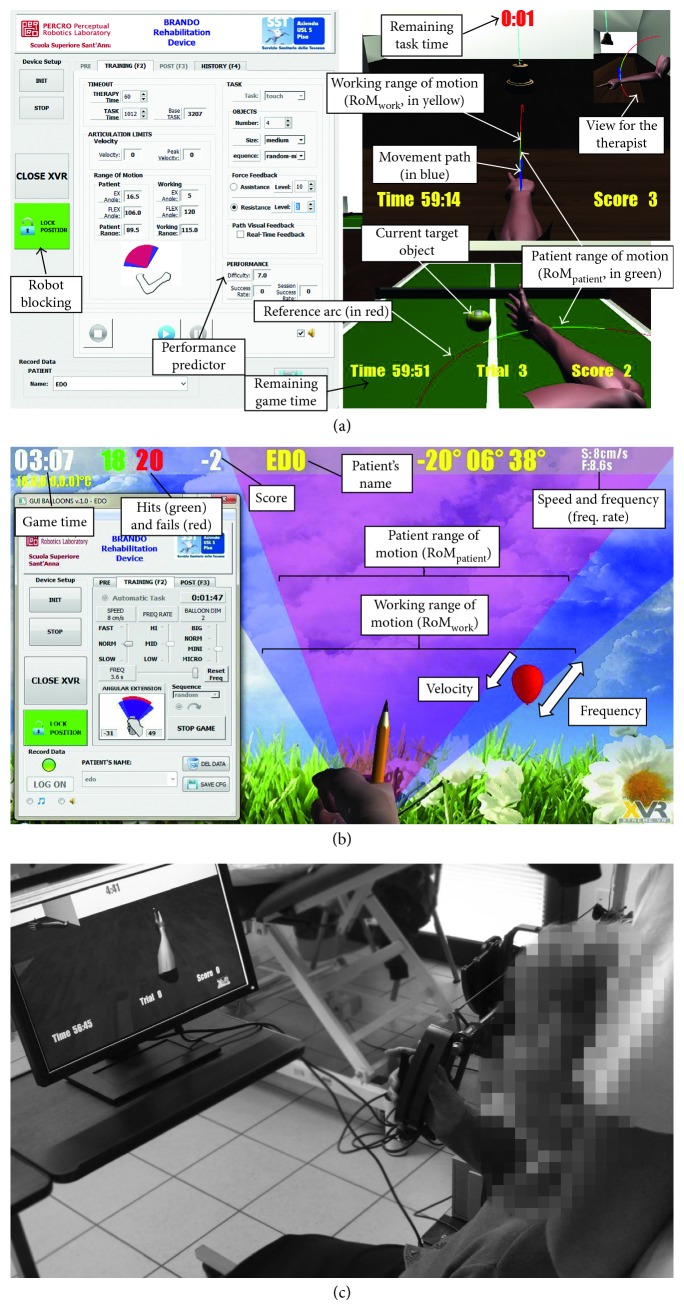
Three views of the full system with the Therapist GUI console and the gaming scenarios with different implemented tasks for the training. (a) FE movements; (b) PS movements; (c) a patient performing with the system.

A software application with VR game scenarios designed explicitly for elbow's FE and wrist's PS motion tasks was developed [36]. Two haptic feedback modes were considered: free PS movements with weight support, while variable force assistance/resistance for FE movements. The software provides the therapist with a user-friendly graphical user interface (GUI) (developed in Python) which allows the therapist to intuitively set the parameters of the current session (speed, the range of movements, and workloads), to select the training modality (passive/active), manually calibrate, and personalise the exercise parameters (Figure 5). After the physiotherapist has chosen the training modality (passive/active) and the game parameters, the system allows him/her to monitor the kinesiology patients' performance (RoM, joint angular velocities, and tolerated haptic force intensities), as well as the patients' achievements regarding game exercises (score, time for completing the task, and the achieved difficulty level). At the end of each session, it could be possible also to generate a report with graphs and statistics corresponding to the patients' evolution (knowledge of performance).

#### 2.4.1. Session Calibration

The session starts with the evaluation of the current motion capacity of the patient. First, the patient is sitting in the correct posture in front of the screen, as explained in Section 2.2. Second, the therapist sets up the lengths of the patient's upper limb and calibrates the training movements. For FE, the calibration is defined by $M{\left(q_{4}^{\mathrm{EX}},q_{4}^{\mathrm{FLEX}},{\bar{\dot{q}}}_{4},{\dot{q}}_{4}^{\mathrm{peak}},t_{\mathrm{base}},K_{p}^{\mathrm{EX}},K_{p}^{\mathrm{FLEX}},F^{\mathrm{EX}},F^{\mathrm{FLEX}}\right)}_{\mathrm{patient}}$, where *q*_4_^EX^ and *q*_4_^FLEX^ are the maximum extension and flexion angles of the elbow, ${\bar{\dot{q}}}_{4}$ and ${\dot{q}}_{4}^{\mathrm{peak}}$ are the mean and peak angular velocities, *t*_base_ is the amount of time for performing movements within RoM_patient_=[*q*_4_^EX^, *q*_4_^FLEX^], and *F*^EX^ and *F*^FLUX^ are the tolerated interacting force amplitudes that the robot will exert during the training. The therapist manually adjusts the gains *K*_p_^EX^ and *K*_p_^FLEX^ of the PD controller, in a way that the observed ${\dot{q}}_{4}$ relies on the range $\left[{\bar{\dot{q}}}_{4},{\dot{q}}_{4}^{\mathrm{peak}}\right]$. For security, *K*_p_^EX^ and *K*_p_^FLEX^ are constrained to be incrementally tuned up by iteratively testing the force step by step within a range of [0, 0.2*n*]Nm/rad, where *n*=1 … 10 is the applied *n*-test by the therapist on the GUI.

For PS, the calibration is defined by $M{\left(q_{5}^{\mathrm{PRON}},q_{5}^{\text{SUP}},{\bar{\dot{q}}}_{5},{\dot{q}}_{5}^{\mathrm{peak}}\right)}_{\mathrm{patient}}$, where *q*_5_^PRON^ and *q*_5_^SUP^ are the angular limits of the pronation and supination movements, and ${\bar{\dot{q}}}_{5}$ and ${\dot{q}}_{5}^{\mathrm{peak}}$ are the mean and peak angular velocities of the wrist during PS.

#### 2.4.2. VR Games for Motion Recovery

Three kinds of movements commonly used in manual therapies were selected: (1) FE with the upper arm adducted (*exercise 1*); (2) FE with the upper arm abducted (*exercise 2*); and (3) PS with the arm adducted (*exercise 3*). Correspondingly, three different VR exergaming scenarios were created (Figure 5). The scenarios simulate different virtual tasks to complete through repetitive movements (Table 1). A scene for touching and ringing a bell with the index finger is for *exercise 1* (scenario *Bells*; Figure 5). A scenario for hitting a tennis ball on a table is for *exercise 2* (scenario *Balls*; Figure 5). A scenario for *exercise 3* is avoiding collisions with balloons gradually getting closer to the virtual hand by bursting them by orienting the pointer through wrist PS movements (scenario *Balloons*; Figure 5).

With the aim to motivate the patient to perform challenging movements and to sustain his/her attention and interest, the difficulty level of the training may increment over the sessions. At the end of the session, the therapist may assess the observed patient's performance; then, the current calibration and game parameters may be used as a baseline for the calibration of the next session and for historical comparison of the patient evolution.

For this purpose, the GUI allows the therapist to manually modify the game (Figures 5 and 5) and consequently the demanding working load level, at any time during the training. To aid the therapist to systematically individualise the training as a function of the game input parameters, a predictive model of patient performance indicates in the GUI the expected performance score (normalised difficulty level from 0 to 10, as detailed in Section 3.4.2).

For FE movements, the following game parameters define the difficulty of both *Bells* and *Balls* exergames, as illustrated in Figure 5:

1. The training workspace RoM_work_ within the range of [1.0, 1.5] times the current patient RoM_patient_
2. The virtual object's *size*
3. The timeout (*t*_task_) for completing the task by scaling [0.5, 1.5] the registered *t*_base_ value
4. The *number of positions* of objects within RoM_work_
5. The *sequence* of appearance of objects at a random position (*random sequence*) or at an arbitrary location in alternate sequences of the extension and flexion (*random mirror sequence*)
6. The exerted haptic forces Force_work_ by scaling the impedance gain *K*_p_ proportionally to the calibrated gains (*K*_p_^EX^ or *K*_p_^FLEX^ for extension and flexion, resp.), within a range of [0, 1] for the assistance feedback and [0, 0.5] for the resistive one

For PS movements, the difficulty parameters of *Balloons* game are the following, as illustrated in Figure 5:

1. The game workspace RoM_work_ beyond RoM_patient_.
2. The *speed* and *size* of the balloons.
3. The *sequence* of appearance of balloons in an *ordered sequence*, at *random* within RoM_patient_, or farther within the lateral cones defined by the intersection RoM_work_∩RoM_patient_.
4. The *frequency rate* of the balloons progressively rises as the number score of the patient increases.

Figure 5 shows a patient performing a rehabilitation session with the BRANDO system while receiving supervision of a physiotherapist.

## 3. Experimental Assessment of System Feasibility and Performance

### 3.1. Objectives of the Study

The objective of the study was to bear out the suitability of the system to be used as an aided method for orthopaedic physiotherapy of the forearm involving both PS and FE joint movements. To this aim, we investigated the following three aspects:

1. The capability of the system to assign different levels of physiotherapy exercises of the forearm, involving both PS and FE joint movements; thus, to elucidate whether, despite the variable working loads, the training remained under controlled and moderate motion conditions according to the current clinical condition of the patient, to avoid any harmful movement, as a fundamental requirement in orthopaedic rehabilitation.
2. The effects of the variable difficulty training conditions on the kinesiologic patient's performance (demanding incremental movements regarding higher ranges of motion, opposite force resistance, and speed) and its possible relationship with their current clinical condition; then, to design a new predictive model of the patient's progress, as a tool for the therapist for individualising the training intensity.
3. The evaluation and acceptance of the system by patients.

For these purposes, the experimental procedure was separated into two parts: (i) a preliminary evaluation of the functionality of the system in healthy subjects and (ii) a pilot study with patients undergoing manual physiotherapy due to forearm lesions.

### 3.2. Recruitment and Patient Population

Two healthy volunteers and ten patients (six males and four females; 47.20 ± 20.47 years old) were recruited at the USL 5 Rehabilitation Centre at Fornacette (Pisa), Italy. Nine performed PS training, while seven performed both PS and FE movements. All patients received a medical indication of the following traditional rehabilitation physiotherapy of the forearm due to fracture(s) at the elbow or wrist joints and after at least a period of 7 to 10 days after the splint withdrawal. None presented fragments instability, severe pain sensation, kinaesthetic or tactile sensorial disorders in the upper limb, or cognitive impairment. The subjects were informed regarding the aspects of the study and signed their informed consent before the experimental sessions. The study was reviewed by the local ethics committee.

All patients underwent a battery of clinical assessments: (i) the ranges of motion with extendable goniometers and following standard procedures [7], (ii) the strength of the affected hand by the Jamar strength test [48], and (iii) the pain sensation using the VAS pain test (Visual Analogue Scale for the pain test) [49]. The musculoskeletal ability to perform activities of daily life applying the Italian version of the DASH Questionnaire (Disabilities of the Arm Shoulder and Hand Questionnaire) normalised to a scale from 0 to 100 where the zero score means no impairment and proper functionality, while 100 means severe impairment and limited functionality [50]. The patients presented reduction of mobility at the limits of functional FE RoM (90.17 ± 23.08°, with 104.25 ± 28.89° of flexion below a functional range of 130° [51] and 13.83 ± 18.48° of extension) and also at the limits of functional PS RoM (118.60 ± 11.07°, with 58.80 ± 20.89° of pronation and 59.80 ± 20.27° of supination), presented a small registered hand strength of 15.40 ± 16.09 kg, reported mild pain sensation of 4.3 ± 2.0, and self-perceived disability of 59.46 ± 10.79% according to the DASH score.  Table 2 shows the clinical characteristics of the patient population.

### 3.3. Preliminary Evaluation in Healthy Subjects

With the first aim of evaluating the functionality of the system, we recruited two healthy volunteers for testing the system before the evaluation in patients. We were particularly interested in studying the capability of the system for assigning incremental working profiles at moderate loads, under safe conditions at any moment and without overloading the patients.

We analysed their muscular activity to verify the effectiveness of the system in assigning different working loads in a training session under similar conditions, as for patients.

The subjects were invited to perform 45 minutes of training with the *Bells* game. Three motion velocities (low, medium, and high), under three haptic feedback modalities, were applied: zero force (ZF), assistance force (AF), and resistance force (RF) feedback. Two different force intensities were used for AF and RF, for a total of 15 working load combinations: 3 velocities *x* (2 AF + 2 RF + 1 ZF). The velocity levels for FE were 80°/s (low), 110°/s (medium), and 190°/s (high) (corresponding to movements within an RoM of 125° in time periods of 1.5 s, 0.9 s, and 0.65 s, resp.). The force feedback consisted of estimated mean values of 2.73 N (medium) and 5 N (high) for AF, while 1.8 N (medium) and 2.5 N (high) for RF. The ZF + medium velocity condition corresponded to natural movements during the calibration at the beginning of the session.

The muscular activation was monitored through surface electromyography (sEMG) by seven pairs of surface electrodes placed on two muscles of the subjects' upper limb: the biceps brachii (BB) and triceps brachii long head (TBL). SENIAM recommendations were followed for sensor positioning and the skin preparation (http://www.seniam.org) [52]. Ag/AgCl foam pregelled electrodes with a diameter of 24 mm were used with an interelectrode distance set to 20 mm for each bipolar derivation. The ground and the reference electrodes for all bipolar derivations were positioned at the elbow. All electrodes were connected to an amplifier (g.USBamp amplifier; http://www.gtec.at/) and digitally converted (1200 Hz sample frequency, 12-bit resolution). We preprocessed the envelopes of the activation signals for analysing isolated movements, as follows.

**Figure 6:**
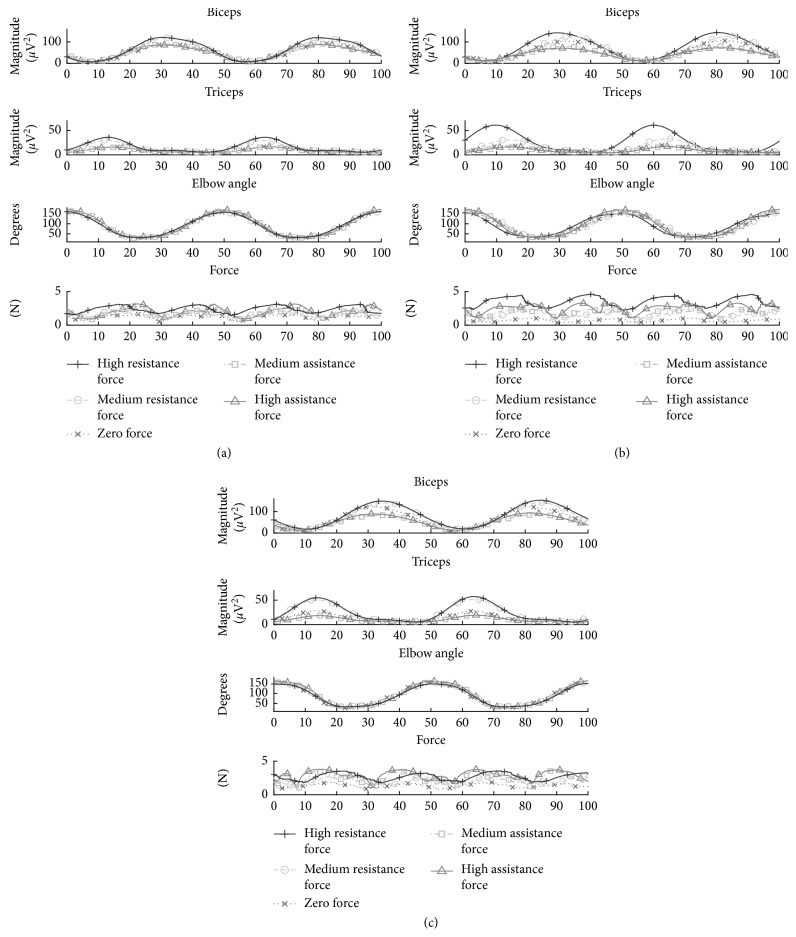
Postprocessed sEMG signals of the monitored muscular activity from the BB and TBL muscles of a healthy subject. The curves represent the median activations of different working load profiles of 3 force levels (resistance, zero or no force, assistance) × 3 movement's speed levels: (a) low, (b) medium, and (c) high.

A band-pass filter was applied (5–500 Hz bandwidth), followed by high-pass filtering (cutoff frequency of 20 Hz), full-wave rectification, and low-pass filtering (1 Hz cutoff frequency). Then, the signals were divided into epochs using the maximum peak of the recorded FE elbow angle as a reference trigger and resampled using a cubic spline interpolation.  Figure 6 shows the average muscle profiles of a healthy volunteer under the different tested conditions.

### 3.4. Pilot Tests in Patients

To verify the suitability of the system to be used as an aided method for orthopaedic physiotherapy of the forearm, we carried out a second experiment with patients. The tests were done to confirm the capability of the system for providing controlled and moderate motion tasks with patients, after the preliminary evaluation with two healthy volunteers. To this purpose, first, we analysed the kinesiologic patient's performance and the relationship of the variable difficulty training levels with the current patient clinical conditions according to the standard clinical outcomes (JAMAR, VAS, and DASH tests). We were also interested in two other issues: (i) the possibility of introducing a new predictive metric of performance as a critical tool for guiding the recovery by the therapist and (ii) assessing the system and verifying its acceptance by patients.

#### 3.4.1. Experimental Session

The tests lasted 30 minutes of exercising divided into three parts, with two pauses of 2 minutes for resting, for a total of 45 minutes per session, including the initial calibration phase. For practicality, for FE movements, all tested the *Bells* game because it did not require the external support of the arm.

Before starting the session, the patient was assisted to sit down in the correct posture. Next, we evaluated the current motion capability (RoM, velocity, and force) of the patient and then calibrated the starting difficulty level of the game. The difficulty was progressively adjusted by the hand during the session, always within safe tolerances. In other words, with the aim of preventing any manual error by the therapist, the system constrained the game parameters to ranges that matched from half to the full current capacity of the patient according to the calibration. In particular, for FE, completion task time *t*_task_ was constrained up to 2*t*_base_, maximum force intensity up to half of the tolerated force intensity (through the impedance gain *K*_p_ up to 1/2*K*_p_^EX^ or 1/2*K*_p_^FLEX^), and RoM_work_ up to 1.2 RoM_patient_. For PS, RoM_work_ was constrained up to 1.2 RoM_patient_. Then, patients were invited to perform and were kindly instructed to concentrate and to express if they felt pain or discomfort during the exercising.

#### 3.4.2. Predictive Model of Patient Performance

With the aim of aiding the therapist to manually individualise the physiotherapy by systematically incrementing the training demand levels, we implemented a model of the *performance* of the patients. For this purpose, a principal component analysis (PCA) was applied to the observed kinesiology information of patients. The model resulted in the linear combination of a performed range of motion, velocity, and tolerated exerted resistive force by the system (RoM_performed_, Velocity_performed_, and Force_performed_) for FE, while RoM_performed_ and Velocity_performed_ for PS. Then, for providing a prior estimation of *performance*, predictive regression models of normalised outcomes of RoM^∗^_performed_ and Velocity^∗^_performed_ for FE and PS and Force^∗^_performed_ for FE were developed, all regarding game input parameters, as follows:

(4) $$\begin{array}{c} \text{Outcome}=b_{o}+\overset{p}{\sum }b_{i}x_{i}+\overset{q}{\underset{j\neq k}{\sum }}b_{jk}x_{j}x_{k}, \end{array}$$

where *x*_*i*_ is the *i*th input parameter among the *p* significant input variables (significant main factors) for the corresponding outcome, *x*_*j*_ and *x*_*k*_ correspond to significant interacting factors, and *b*_*n*_ are constant values. Significant main factors and interactions over outcomes (RoM_performed_, Velocity_performed_, and Force_performed_) were identified through a series of multifactorial ANOVAs (MANOVAs). The MANOVA series followed a design of 3 target *sequences* × 3 levels of *t*_task_ × 3 levels of RoM_work_ × 3 levels of Force_work_ for FE movements, while 2 *sequences* × 3 RoM_work_ × 3 *balls' number* × 3 *balls' speed* × 3 *balls' frequency* for PS movements.

Finally, optimisation of the regression models was carried out over the training data set of 7 patients for PS and six patients for FE, while we used the data of 2 patients for PS and one patient for FE for model comparison and validation.

#### 3.4.3. System Acceptance Evaluation

We applied an ad hoc designed questionnaire with eight items, all rated on a 7-point Likert scale (where 1 was the minimum, 7 the maximum, and 4 the neutral scores), for qualifying diverse perceived aspects regarding the confidence and acceptability of the system (Table 3). Six items assessed the usability and acceptance of the system: (1) How much the patients perceived it enjoyable (*enjoy*)? (2) How much difficult it was (*difficult*)? (3) How much exhausting they felt (*fatigue*)? (4) How much attention they paid (*attention*)? (5) How much physical pain patients felt in the affected limb during the exercises (*pain*)? (6) How much annoyed they felt during the exercises (*annoyance*)? Two extra items evaluated the embodiment sensation during the sessions [45, 53], regarding two sensations: (1) the sense of *ownership*, which means how much they perceived that the virtual avatar was their own limb, and (2) the sense of *agency* that indicates how much they recognised that the movements and actions of the virtual avatar were caused by their own actions.

## 4. Results

### 4.1. Preliminary Evaluation in Healthy Subjects

**Figure 7:**
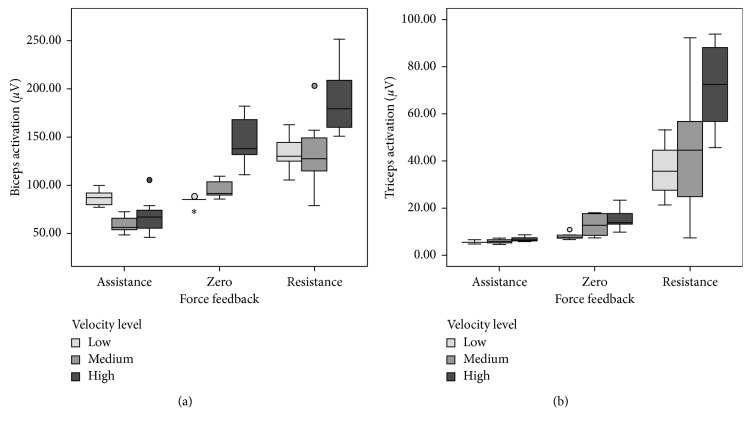
Observed amplitudes of the postprocessed muscular activation signals of the isolated flexion/extension elbow's movements for the 3 force × 3 velocity conditions. Main effect factors and significant interactions for force and velocity on both BB (a) and TBL (b) muscles.

Different activation levels were observed for both BB and TBL (Figure 6), being the biggest motor activation of arm muscles associated with the movements performed under resistance forces, following by free movements (zero force) and those with the lowest level of assistance forces. We also observed decreasing motor activations from high to medium and from medium to low velocity movements. The increments in motor activation were due to dynamic isometric contractions during voluntary active movements under assistance or resistance forces and revealed by the increase of the amplitude of the sEMG [54]. A repeated measures ANOVA (SPSS 15 statistical package) according to 3 forces × 3 velocity conditions over mean amplitudes of the signal envelopes (Figure 7) revealed main factor effects for both force (*F*(2, 10) = 97.069, *p* < 0.05) and velocity (*F*(2, 10) = 26.872, *p* < 0.05) and a significant interaction (*F*(4, 20) = 12.222, *p* < 0.05) for BB, and similarly for force (*F*(2, 10) = 606.725, *p* < 0.05) and velocity (*F*(2, 10) = 24.106, *p* < 0.05) and a significant interaction (*F*(4, 20) = 16.037, *p* < 0.05) for TBL, which confirmed the recruitment of higher motor units as the system increments the required resistance force and velocity of movements, as expected. No significant changes in the mean frequency of the signals were observed, which indicates variable muscular working load at the arm while flexing and extending the elbow with the system but without overloading it until excessive muscle fatigue [55].

### 4.2. Pilot Tests in Patients

For assessing the capability of the system for estimating the range of motion of the patients' movements, a comparative analysis was carried out between the standard clinical RoM outcome manually estimated by the therapist with the manual goniometer (Table 2) and the online estimation performed by the system (RoMpatient) during the calibration step at the beginning of the session (Table 4).

For FE RoM, an underestimation was observed, from a mean clinical RoM of 101.57 ± 6.32° for the seven patients performing FE training, in comparison to the mean calibrated RoM_patient_ = 92 ± 10.63°. An error of 11.66 ± 10.69% was observed between both measures, with a medium value of the Pearson correlation of *r* = −0.278, but not still significant given the small sample size. For PS, a mean clinical RoM of 118.60 ± 11.067° was observed among patients, while a mean calibrated RoM_patient_ = 117.90 ± 10.59° was observed with an error of 5.66 ± 6.64% and a high value of the Pearson correlation of *r* = 0.520 but again not still significant.

An interpretation of the observed errors is that the estimation of RoM_patient_ during the calibration phase of the training session is consistent with the clinical observation and valuable for personalising the level of the working load difficulty of the training and the scoring of the estimated performance as a function of real-time kinaesthetic information. However, on the contrary, the RoM_patient_ estimation must not be considered as a valid clinical measure that may replace the current standard manual method.

During the experiments, we incremented the difficulty levels from the observed motion capacity of the patients after the calibration phase. The increments included force resistance levels and working range of motion, decrements in the task's time, increasing number of targets, and different sequences of targets' position. Patients were invited to test the system just one session, so we expected that, at first sight, patients would probably perceive the system with suspiciousness. For this reason, the game parameters were manually adjusted to maintain the achieved game score as high as possible while keeping safe movements' conditions, which resulted in the observed performance in Table 4.

We carried out a correlation analysis between the observed kinetic information of patients during the training and the outcomes of current clinical assessment (JAMAR, VAS, and DASH scores) to elucidate any possible relationship between the patient's clinical condition and the difficulty game conditions.

First, the patient strength given the JAMAR score was found to be negatively correlated (*r* = −0.615) with the mean executed RoM during the game (*performed FE RoM*; Table 4). The contrary was less correlated with the mean working load (*FE load*, *r* = 0.126) and velocity of movements (*FE velocity*, *r* = −0.172). These correlations can be explained by the fact that even though the achievable range of motion could infer muscular weakness, muscular weakness is not ultimately expressed in the dynamic components of movements (loads and velocity) since for safety requirements, the working loads were calibrated for assuring moderate levels of motion. A moderate negative correlation (*r* = −0.369) between the pain sensation (VAS score) and the performed RoM during FE training (*Performed FE RoM*) indicates that the patients who reported higher pain sensation were more cautious of performing painful movements, and thus, they performed smaller RoM. The small correlations found between VAS scores with both working loads (*FE load*) and velocity of movements (*FE velocity*) confirm that the rehabilitation sessions were carried out at moderate dynamic levels.

Regarding the relationship of the self-perception of the ability to perform daily-life activities through the DASH score, we found a positive correlation with the *performed FE RoM* (*r* = 0.417) and a negative correlation with *FE load* (*r* = −0.468). Both correlations highlight that the kinesiological performance of patients was lower in those patients showing higher current disabilities than in patients with less daily-life difficulties, as occurred with patients P3, P5, and P7 who suffered from injuries affecting elbow FE. It is important to notice that patient P1 expressed a high pain sensation in the VAS test but did not show difficulties in *performed FE RoM* because of the primary suffering that was in PS instead of FE movements.

Regarding the PS movements, negative correlations were found between *performed PS RoM* with the DASH score (−0.439) and with the JAMAR score (*r* = −0.372). An explanation to the observed correlations is the fact that the higher the level of impairment, the more the difficulty to perform the rehabilitation exercise involving PS movements.

In general, the working RoM_work_ was set to be slightly higher than the patient's one (measured during the system calibration). Consequently, the speed of the objects on the screen was set for resulting in mild slow motions (FE and PS velocities) within the performed RoM. For FE, the adaptive loads assigned from zero up to 0.5 kg were well tolerated by the patients. A necessary clarification at this point is that working load for patients remained under the same range applied to healthy volunteers, as can be observed in the force feedback profiles in Figure 6 (with values up to 5 N, equivalent to 0.5 kg loads).

During all the tests, we did not observe any unexpected event that may cause risk to the patients. Moreover, all patients remained calm during the sessions and did not perceive or express any threat due to the system.

Then, with the aim of modelling the kinematic performance score, a PCA was implemented over the training data sets of six patients for FE and seven patients for PS to perform dimension reduction of the data and obtain a metric of *performance* as a linear combination of the normalised values of the observed RoM_performed_, Velocity_performed_, and Force_performed_.  Table 5 shows the coefficient values of the two principal components: for FE, the first and second components explain 62.38% and 29.57% of the variance, respectively, while for PS, the first and second components explain 68.44% and 31.56% of the variance, resulting in the *performance* equations in Table 5.

Then, for FE movements, the series of MANOVAs over outcomes revealed significant main factor effects of RoM_work_ (*p* < 0.0001) and a significant interaction of *t*_task_ ∗ RoM_work_ (*p* < 0.007) for the observed RoM_performed_. For the observed Velocity_performed_, main factors were found for *t*_task_ and RoM_work_ (*p* < 0.0001) with no significant interaction. For the observed Force_performed_, main factors were found for *t*_task_ (*p*=0.05), Force_work_ (*p* < 0.0001), and a significant interaction of *t*_task_ ∗ Force_work_ (*p* < 0.0001). On the contrary, for PS movements, main factor effects of RoM_work_ (*p* < 0.0001) for RoM_performed_ were revealed; for Velocity_performed_, main factor effects of *sequence* (*p* < 0.0001), balls_frequency_ (*p* < 0.0001), and balls_speed_ (*p* < 0.024) and a significant interaction between *sequence* ∗ balls_number_ (*p* < 0.01), *sequence* ∗ balls_speed_ (*p* < 0.032), and RoM_work_ ∗ balls_frequency_ (*p*=0.05) were revealed.

**Figure 8:**
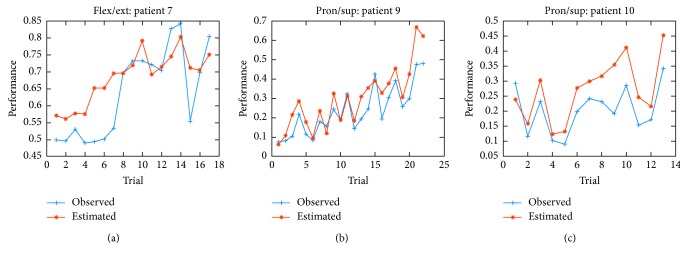
Comparison of the performance (from 0 to 1) between the predicted and observed score for the group of three testing patients: (a) patient 7 for FE, and (b) patient 9 and (c) patient 10 for PS movements.

Consequently, the predictive regression models given the equations in Table 6 were obtained, as a function of the difficult input parameters estimated over the corresponding training data. The regression models presented a significant correlation of *r* = 0.837 (the Pearson coefficient) comparing the predicted and observed performance over one patient data for FE and *r* = 0.917 over two patients data for PS. This correlation indicates a good agreement between the estimated performance during the setting up of the difficult training parameters and the observed performance during the training under such parameters, as shown in Figure 8.

**Figure 9:**
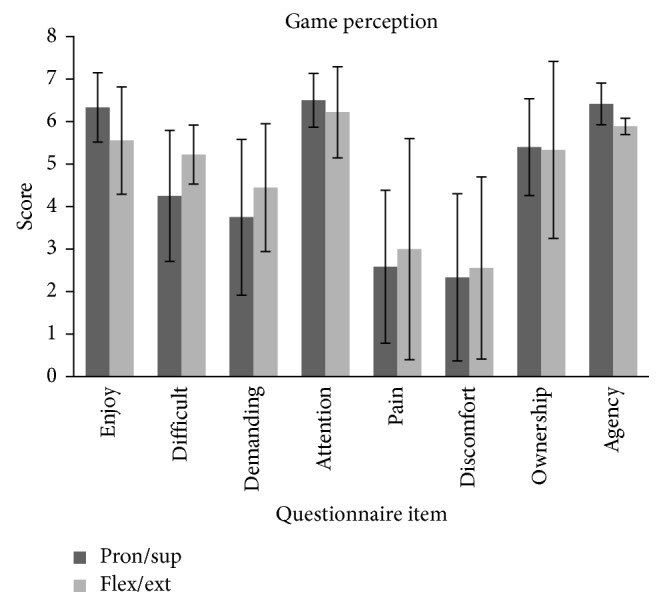
Observed values of the self-reported scores of the ad hoc questionnaire for assessing the perception and opinion of the system by the patients. Items *enjoy, difficult, fatigue, attention, pain,* and *annoyance* evaluated the confidence and acceptance of the system, while *ownership* and *agency* assessed the embodiment experience during the sessions.

Figure 9 shows the observed scores of the self-rated questionnaire for assessing the opinion and experience of the patients about the system. We adopted the criteria of considering a median score ≥5, as the indication that the diverse aspects were experienced highly. Patients found the games enjoyable (median of 6.50 ± 0.82 in *enjoy* for PS and 5.50 ± 1.26 for FE). They experienced neutral difficulty (4.50 ± 0.82 for PS and 5 ± 0.69 for FE in *difficult*) and neutral *fatigue* (4.00 ± 1.97 for PS and 4.50 ± 1.50 for FE), and the sessions were enough demanding for keeping their *attention* quite high (6.75 ± 0.75 in *attention* for PS and 6.50 ± 1.07 for FE). Regarding the perceived side effects, most patients expressed feeling some mild *pain* (2.00 ± 2.02 for PS and 2.50 ± 2.60 for FE) and low *annoyance* (1.50 ± 2.16 for PS and 2.50 ± 2.14 for FE). High embodiment sensation of the system reflected in the high scores for the *ownership* (5.00 ± 1.29 for PS and 5.00 ± 2.08 for FE) and *agency* (6.25 ± 0.50 for PS and 6 ± 0.19 for FE) sensations over the virtual avatar. Finally, at the end of the session, besides the questionnaire, they verbally express their high interest in participating in a more extended clinical study with the system.

## 5. Discussion

The present work has three important aspects: (i) the development for the first time of a robotic-VR-mediated application specific for the orthopaedic rehabilitation of the forearm, (ii) the development of a predictive model of patient performance for aiding the individualisation of the training exercises regarding observed kinesiology information, and (iii) an evaluation of the system by two healthy subjects and a group of patients to study its acceptance and the feasibility of carrying out clinical studies.

The system was specially developed for covering the clinical needs of orthopaedic rehabilitation of injured upper limbs affecting the forearm mobility, similarly as the manual method. Diversely from current neurorehabilitation robotic systems, in this approach, three essential aspects were considered of particular interest in orthopaedics: the recovery of functional range of motion [9, 51], muscular strength, and pain reduction [5, 49]. The system integrates some elements already reported separately for neurorehabilitation, such as resistive [26] and assistive force fields and jerk trajectories [25], but combined and adapted in a new methodology that satisfies the particular needs of isometric passive, active, or active-assistive motion training of single joints. An important characteristic is that it allows the therapist to assign different training loads, imposing incremental motion tasks starting from small training arcs and mild force loads up to a painless normal range of motion and moderate force loads.

The restoration of functional ranges of motion is constrained by the system to be progressively incremented with small steps, during all the assisted treatment process, in particular for the flexion/extension arc from the current ranges up to a minimum functional range of motion of 100° [51] and for pronation/supination up to a functional range of 50° [9, 51]. In this way, the system guarantees the gradual and careful tuning up of the task motion requirements to the patients. Slight increments in working loads are also possible through small increases in the intensity of the force feedback, but at any moment up to moderate tolerated levels observed during the calibration phase at the beginning of each session. This characteristic is crucial to avoid harmful movements that may result in severe side effects such as destabilisation, inflammation, and oedema or time delay in the rehabilitation process, with the consequent risk of developing joint stiffness and contracture.

In the field of neurorehabilitation, a current trend is the development of adaptive methods for the individualisation training with the aim of the optimisation of neuroplasticity and motor relearning [28, 56, 57]. In the scope of orthopaedic rehabilitation, this would play an essential role in stimulating the patient to perform progressively more challenging movements to promote the motivation and adherence to the treatment and is more important as a crucial tool in modulating the incremental kinesiologic requirements of patients through time. To this aim, the system includes a predictive model of kinesiologic performance which scores the patient evolution as a function of the online range of motion, force feedback intensity, and velocity of movements. This module enables the possibility to manually personalise the difficulty level of the therapy during the game in a systematic quantitative manner. This information is also useful for managing and reporting online and historical data of the patient evolution during the treatment by the therapist.

Regarding the evaluation of the system, the observed increments in amplitude in the sEMG signals of the volunteers showed that the system allows assigning significant different levels of working loads, due to the combination of required strength and velocity. The analysis over the mean amplitude and mean frequency of the sEMG signals confirmed that the different loads effectively demanded different levels of motor unit activations at the arm muscles, but for safety always keeping the activations below muscular fatigue [54, 55].

The observed mean force feedback intensity remained similar for both healthy participants and patients (below the equivalent load of 0.5 kg). Moreover, since the resulting training profiles of the two healthy volunteers corresponded to profiles conceived for patients, we argue that the working load may be modulated safely by the therapist, according to the observed progress of the patient during the treatment. At this point, we also reviewed the relationship of the observed kinesiology metrics and the current clinical outcomes (VAS, JAMAR, and DASH), and due to the observed correlations between metrics and the clinical scores, we may conclude that the motion tasks were programmed efficiently from mild to moderate demanding.

In fact, the results of the experimental sessions indicate that it is possible to modulate the expected patient performance during the sessions, as a function of kinesiology metrics computed in real time, through the combination of difficulty game parameters. The metrics consider the achieved range of motion, the velocity of movements, and the tolerated opposing forces for the flexion/extension movements, while the range of motion and the velocity of movements for pronation/supination. Moreover, the implemented prediction model of performance shows a good agreement between the estimated scores before and during the training, indicating that it is possible to objectively assign therapeutic levels systematically, which we argue may play an essential role for the individualisation of the therapy and the optimal evolution through the treatment.

The questionnaire for assessing the system revealed that all patients enjoyed playing the games and that the assigned difficulty levels were well balanced and demanded their high attention, which for motor improvement through cognitive activities is crucial. The questionnaires also confirmed that patients felt a mild perception of pain, which is line with the reported scores with the VAS test. Interestingly, patients experienced a high embodiment sensation of the virtual representation of their limb in the scenarios; patients were able to see their forearm, but still perceived the sensation of ownership of the virtual representation of their injured limbs [58]. This fact could be valuable for the incorporation of new proprioceptive exercises and as a biofeedback method that helps the patients to be aware of their current physical limitations, such as abnormal movement synergies, compensatory movements, and limited mobility, among other aspects difficulty to perceive at first sight during daily activities. Moreover, this issue could be valuable to design new games more related to daily-life situations, promoting the (re)embodiment of the injured limb in their body schema, especially during the early mobilisation stage.

Patients perceived slightly more difficult the game for FE than for PS movements because they performed free movements for PS with the forearm static, while for FE variable force-resistant movements for placing the hand at different positions in space.

The experimental sessions were designed in the same conditions that would be applied during robotic-assisted physiotherapy sessions. During the tests, we incremented the difficulty levels from the observed motion capacity of the patients after the calibration phase. The increments include force resistance levels and working range of motion risings, decrements in the task's time, increasing number of targets, and different sequences of position targets. No matter the difficulty levels, we did not observe any unexpected event that may cause risk to the patients. Moreover, all patients remained calm during the sessions and did not perceive or express any threat generated by the system, even when experiencing more challenging conditions.

Since the experimental conditions were designed to be applied similarly to sessions during the assisted therapy and we did not observe risk situations during the tests, we are confident that the system was well accepted. Moreover, we conclude that the system is safe enough to clinically validate it in an interventional study, as a next research step shortly.

However, regarding the estimated ranges of motion of patients with the system at the beginning of the session, we observed an underestimation concerning the standard clinical outcome. A factor that may affect the precision of the inverse kinematic algorithm is that, for computing the joint angles of the upper limb from the wrist position in space, a fixed posture of the shoulder is assumed, so avoiding slight trunk adjustment postures by patients bias the estimation. Additionally, the algorithm also depends on input parameters such as the dimensions of the arm and forearm, so manual errors in such measures may also influence the inaccuracy. Consequently, the range evaluation must be considered as an indicative measure of kinesiologic performance during the training, but not as a valid clinical value. Therefore, it would be desirable to incorporate other technologies such as wearable sensors [59, 60] for the precise assessment of other kinetic aspects such as compensatory movements.

Another limitation is that the current state of the system does not enable the possibility of force feedback during the pronation/supination treatment like other systems conceived for orthopaedic rehabilitation [35–37], which may reduce the chances of shortening the recovery period because of the impossibility to assign variable resistant exercises. By fortune, even if the effects of pronation/supination recovery cannot be neglected for strength recovery, it accounts in less extent than elbow strengthening training [33], so our system can be still a significant tool for aiding the rehabilitation process of the forearm. Since the robot design followed and end-effector based approach, instead of an orthotic one [14], the system is unable to impose strict, joint constraints mechanically. So is not feasible for the very first period of the physiotherapy after immobilisation; in this case, not before seven days after the withdrawal of the splint immobiliser. Therefore, even if the system enables the possibility of providing small assistive forces to the patient, we figure out from the experiments that this feature has no practical clinical use. On the contrary, it would be more helpful to provide small-to-moderate resistive forces after the first week of manual mobilisation.

A third limitation is that, for the moment, the kinesiology performance calibration corresponded to pilot tests with patients performing mild exercising, as observed with high games scores (hits versus failures), which would lead to biases in the estimated performance probably below optimal intensities levels. Moreover, since the hits/failure rates in the game may involve cognitive abilities and not necessarily kinesiology performance, it is not still clear if there is a direct correlation between game and kinesiology scores that would lead in mechanisms for balancing the physical and cognitive requirements to patients. So, to optimise both their performance and cognitively demanding tasks (involving attention, perception, the complexity of the game, and motivation, among other), more research in nonlinear predictive modelling is required.

Finally, due to the limited number of patients, the statistical results would be interpreted as preliminary evidence of the feasibility of the proposed methodology, but more comparative studies are needed with more patients and healthy subjects for confirming the entire validity of the proposal.

## 6. Conclusions

Rehabilitation robotics offers the possibility of new methods of physiotherapy in orthopaedics with patients with musculoskeletal injuries, such bone fractures. As a study case, here we presented a new approach to assisted orthopaedic rehabilitation method of the forearm, involving the elbow and wrist joints. Our proposal combines an end-effector robotic system and a virtual-reality mediated software application with the capability of delivering passive, active, and assisted exercising training of flexion/extension and pronation/supination of injured elbow affecting the forearm. The proposed methodology exploits some existing methods reported separately for neurorehabilitation but integrated within a new framework conceived explicitly for the orthopaedics clinical goal of recovery of functional range of motion, strength, pain reduction, and stiffness prevention.

We studied the possibilities of personalising the exercise's intensity and modify it manually according to the kinesiologic performance of the patient, within safe and moderate controlled online increments during the games, in a more systematic way than traditional manual physiotherapy. The results of our experiments in healthy participants and patients showed that the proposed strategy is suitable. Besides its limitations, the present work contributed to promoting the development of new assisted methods in orthopaedics and further research in this area. We conclude that the proposed approach may have the potential of enhancing the current manual methods, incrementing the hours of therapy per patients and the number of patients simultaneously and reducing the treatment discharge periods.

Future work involves the validation of the system during interventional clinical studies combining manual and assisted sessions some days per week during the whole duration of treatments. Additionally, the developing of other scenarios and the extension of the current system to other musculoskeletal deficits of the upper limb involving other movements would be valuable and may promote the development of new physiotherapy patient-specific methods in the scope of orthopaedic rehabilitation.

## Figures and Tables

**Table 1:** Design specification of the simulated tasks to perform during training exercises.

Exercise	Scenario	Upper arm posture	Movement	Joint motion	Virtual task
1	*Bells*	Adducted	Upward/downward	FE	Ring
2	*Balls*	Abducted	Lateral/medial	FE	Hit
3	*Balloons*	Adducted	Internal/external	PS	Burst

**Table 2:** Clinical characteristics of the patients' population of the pilot study.

Patient	Gender	Age	Fracture injury	Injury treatment	Flex (deg)	Ext (deg)	FE RoM (deg)	Pron (deg)	Sup (deg)	PS RoM (deg)	JAMAR test	VAS test	DASH test
P1	M	24	Humerus, radius and ulna	Operative with internal fixation	138	36	102	78	48	109	14	2	71.33
P2	W	87	Humerus, radius and ulna	Operative with internal fixation	135	40	95	22	87	126	0	1	54.33
P3	M	66	Radius, capitate	Nonoperative with a fixed splint	115	10	105	26	95	121	16	4	63.33
P4	W	35	Humerus, radius and ulna	Operative with internal fixation	100	−5	105	70	40	110	0	5	53.33
P5	W	22	Distal epifisi, radius, ulnar styloid, scaphoid	Operative with internal fixation	128	31	97	80	30	110	15	6	47,41
P6	M	42	Humerus	Operative for removal of internal fixation	90	−5	95	78	55	133	48	6	49.17
P7	M	45	Humerus, radius and ulna	Operative with internal fixation	124	12	112	52	55	107	36	7	50
P8	W	60	Epifisi, radius, ulnar styloid, scaphoid	Nonoperative with a fixed splint	—	—	—	68	56	124	5	5	67.50
P9	M	58	Capitate	Nonoperative with a fixed splint	—	—	—	52	57	109	0	5	77.67
P10	M	33	Radius, capitate	Operative with internal fixation	—	—	—	62	75	137	20	2	48.5

**Table 3:** Self-rated questionnaire for assessing the acceptance of the system and the experience of the patient during the robotic session.

Item	Assessment	Statement	Tag
Q1	Acceptance	How much you consider that the session was enjoyable and engaging?	Enjoy
Q2	Acceptance	How much difficult seemed to you the game?	Difficult
Q3	Acceptance	How much you consider your level of fatigue after the session?	Fatigue
Q4	Acceptance	How much attention did you pay during the execution of the game?	Attention
Q5	Acceptance	How much you consider your level of pain after the session?	Pain
Q6	Acceptance	How much you consider your level of annoyance during the session?	Annoyance
Q7	Embodiment sensation	Sometimes I felt that the virtual arm was my own arm.	Ownership
Q8	Embodiment sensation	The movements of the virtual hand and arm were caused by my movements.	Agency

**Table 4:** Observed patients' performance during the game tests.

Patient	Working FE RoM (deg)	Performed FE RoM (deg)	FE velocity (deg/s)	FE load (kg)	FE game score (0–100)	Working PS RoM (deg)	Performed PS RoM (deg)	PS velocity (deg/s)	PS game score (0–100)
P1	73 ± 13	101 ± 6	79.22 ± 22.1	0.27 ± 0.14	88.15 ± 15.5	104 ± 13	78 ± 13	46.80 ± 20.90	77.05 ± 0.24
P2	99 ± 15	91 ± 6	86.14 ± 28.44	0.19 ± 0.16	93.40 ± 7.2	123 ± 12	98 ± 13	87.11 ± 44.17	96.50 ± 0.05
P3	81 ± 22	83 ± 16	66.91 ± 13.57	0.29 ± 0.19	64.30 ± 5.3	113 ± 11	83 ± 12	89.14 ± 53.13	87.12 ± 0.07
P4	99 ± 16	101 ± 6	86.14 ± 28.44	0.19 ± 0.16	93.40 ± 7.2	129 ± 11	111 ± 9	85.56 ± 51.01	94.49 ± 0.09
P5	97 ± 14	91 ± 3	112 ± 45.18	0.44 ± 0.11	81.73 ± 11.3	121 ± 23	99 ± 20	53.79 ± 21.88	93.71 ± 0.067
P6	100 ± 4	84 ± 8	88.32 ± 50.92	0.29 ± 0.14	85.29 ± 15.2	112 ± 14	85 ± 2	78.71 ± 27.90	87.63 ± 0.10
P7	95.17 ± 9.38	87.43 ± 18.5	67.10 ± 14.27	0.175 ± 0.04	88.94 ± 15.8	—	—	—	—
P8						123 ± 6	98 ± 5	54 ± 22	93.71 ± 0.067
P9						123 ± 11	94 ± 11	79 ± 28	87.63 ± 0.1
P10						136 ± 8	112 ± 13	44 ± 22	97.19 ± 4.33

**Table 5:** Results from PCA over observed kinesiology outcomes explaining the first and second components, around 65% and 30% of the variance and defining the *performance* equation as a linear combination of kinesiology information.

Outcome	PC_1_	PC_2_	Performance equation
*Flexion/Extension*			
RoM_performed_	0.285	0.4424	Performance_FE_=0.6238PC_1_+0.2957PC_2_
Velocity_performed_	0.618	0.5929	
Force_performed_	0.7323	0.6729	

Pronation/supination			
RoM_performed_	0.4899	-0.8718	Performance_PS_=0.6844PC_1_+0.3156PC_2_
Velocity_performed_	0.8718	0.4899	

**Table 6:** Regression models for predicting kinesiology information of the achieved range of motion, the velocity of movements, and exerted opposing forces, as a function of difficulty input parameters.

Movement	Regression predictive model	No significant effect for the model (*p* > 0.05)
FE	RoM^∗^_performed_ = *b*_0_ + *b*_1_ ∗ RoM_work_	*t*_task_ ∗ RoM_work_
FE	Velocity^∗^_performed_ = *b*_0_ + *b*_1_ ∗ *t*_task_ + *b*2 ∗ RoM_work_	—
FE	Force^∗^_performed_ = *b*_0_ + *b*_1_ ∗ Force_work_ + *b*_2_ ∗ *t*_task_ ∗ RoM_work_	*t*_task_
PS	RoM^∗^_performed_ = *b*_0_ + *b*_1_ ∗ RoM_work_	—
PS	Velocity^∗^_performed_ = *b*_0_ + b_1_ ∗ sequence + *b*_2_ ∗ balls_speed_ + *b*_3_ ∗ sequence ∗ balls_speed_ + *b*_4_ ∗ RoM_work_ ∗ balls_frequency_	balls_frequency_, sequence ∗ balls_number_
